# Trends in Treatment and Survival of Gallbladder Cancer in the Netherlands; Identifying Gaps and Opportunities from a Nation-Wide Cohort

**DOI:** 10.3390/cancers12040918

**Published:** 2020-04-09

**Authors:** Elise de Savornin Lohman, Tessa de Bitter, Rob Verhoeven, Lydia van der Geest, Jeroen Hagendoorn, Nadia Haj Mohammad, Freek Daams, Heinz-Josef Klümpen, Thomas van Gulik, Joris Erdmann, Marieke de Boer, Frederik Hoogwater, Bas Groot Koerkamp, Andries Braat, Joanne Verheij, Iris Nagtegaal, Cornelis van Laarhoven, Peter van den Boezem, Rachel van der Post, Philip de Reuver

**Affiliations:** 1 Department of Surgery, Radboud University Medical Centre, 6500 HB Nijmegen, The Netherlands; elise.desavorninlohman@radboudumc.nl (E.d.S.L.); R.Verhoeven@iknl.nl (R.V.); kees.vanlaarhoven@radboudumc.nl (C.v.L.); peter.vandenboezem@radboudumc.nl (P.v.d.B.); 2 Department of Pathology, Radboud University Medical Centre, 6500 HB Nijmegen, The Netherlands; tessa.debitter@radboudumc.nl (T.d.B.); Iris.Nagtegaal@radboudumc.nl (I.N.); chella.vanderpost@radboudumc.nl (R.v.d.P.); 3 Department of Research, Netherlands Comprehensive Cancer Organization, 3501 DB Utrecht, The Netherlands; l.vandergeest@iknl.nl; 4 Department of Surgery, Utrecht University Medical Center, 3508 GA Utrecht, The Netherlands; j.hagendoorn-3@umcutrecht.nl; 5 Department of Medical Oncology, Utrecht University Medical Center, Utrecht University, 3508 GA Utrecht, The Netherlands; n.hajmohammad@umcutrecht.nl; 6 Department of Surgery, Amsterdam University Medical Centers, VU University, Cancer Center Amsterdam, 1007 MB Amsterdam, The Netherlands; f.daams@amsterdamumc.nl; 7 Department of Medical Oncology, Amsterdam University Medical Centers, University of Amsterdam, Cancer Center Amsterdam, 1100 DD Amsterdam, The Netherlands; h.klumpen@amsterdamumc.nl; 8 Department of Surgery, Amsterdam University Medical Centers, University of Amsterdam, Cancer Center Amsterdam, 1100DD Amsterdam, The Netherlands; t.m.vangulik@amsterdamumc.nl (T.v.G.); j.i.erdmann@amsterdamumc.nl (J.E.); 9 Department of Surgery, Section of HPB-Surgery and Liver Transplantation, University Medical Center Groningen, 97700 RB Groningen, The Netherlands; m.t.de.boer@umcg.nl (M.d.B.); f.j.h.hoogwater@umcg.nl (F.H.); 10 Department of Surgery, Erasmus MC, 3000 CB Rotterdam, The Netherlands; b.grootkoerkamp@erasmusmc.nl; 11 Department of Surgery, Leiden University Medical Center, 2300 RC Leiden, The Netherlands; a.e.braat@lumc.nl; 12 Department of Pathology, Amsterdam University Medical Center, 1100 DD Amsterdam, The Netherlands; j.verheij@amsterdamumc.nl

**Keywords:** gallbladder neoplasms, cohort studies, chemotherapy, surgery, epidemiology

## Abstract

Gallbladder cancer (GBC) is rare in Western populations and data about treatment and outcomes are scarce. This study aims to analyze survival and identify opportunities for improvement using population-based data from a low-incidence country. GBC patients diagnosed between 2005 and 2016 with GBC were identified from the Netherlands Cancer Registry. Patients were grouped according to time period (2005–2009/2010–2016) and disease stage. Trends in treatment and overall survival (OS) were analyzed. In total 1834 patients were included: 661 (36%) patients with resected, 278 (15%) with non-resected non-metastatic, and 895 (49%) with metastatic GBC. Use of radical versus simple cholecystectomy (12% vs. 26%, *p* < 0.001) in early (pT1b/T2) GBC increased. More patients with metastatic GBC received chemotherapy (11% vs. 29%, *p* < 0.001). OS improved from 4.8 months (2005–2009) to 6.1 months (2010–2016) (*p* = 0.012). Median OS increased over time (2005–2009 vs. 2010–2016) in resected (19.4 to 26.8 months, *p* = 0.038) and metastatic (2.3 vs. 3.4 months, *p* = 0.001) GBC but not in unresected, non-metastatic GBC. In early GBC, patients with radical cholecystectomy had a median OS of 76.7 compared to 18.4 months for simple cholecystectomy (*p* < 0.001). Palliative chemotherapy showed superior (*p* < 0.001) survival in metastatic (7.3 versus 2.1 months) and non-resected non-metastatic (7.7 versus 3.5 months) GBC. In conclusion, survival of GBC remains poor. Radical surgery and palliative chemotherapy appear to improve prognosis but remain under-utilized.

## 1. Background

Gallbladder cancer (GBC) is a rare and highly lethal neoplasm of the biliary tract. GBC demonstrates marked geographic, age-, gender-, and ethnicity-related differences in incidence, implying (epi)genetics or environmental factors may play an important role in the development of GBC [1,2,3,4,5,6]. Other possible risk factors include cholelithiasis, obesity, gallbladder polyps, chronic infections, and an abnormal pancreaticobiliary duct junction [1,7,8].

Treatment of GBC remains challenging. Diagnosis—unless incidentally after cholecystectomy for benign gallbladder disease—is often made in an advanced stage and survival is extremely poor due to the limited efficacy of systemic therapy options [3]. The only treatment with curative intent is surgical resection. However, due to late detection and a tendency towards invasive local growth, only 10% to 25% of tumors are candidates for potential curative intent surgery at presentation [9,10]. Even after resection 5-year survival rates are poor, ranging from 12% to 40% in non-incidental tumors [3,11,12]. Long-term survival is only observed in patients with early (T1/T2) GBC, which is mainly diagnosed incidentally. However, even for these patients, additional radical surgery with resection of the gallbladder bed and lymph node dissection of the hepatoduodenal ligament is recommended because it is thought to considerably increase survival [13,14].

The limited benefit of systemic therapy in GBC has been shown in prospective trials; in 2010, the ABC-02 trial reported a median overall survival (OS) of 11.7 months vs. 8.1 months in unresectable biliary tract cancer treated with gemcitabine and cisplatin versus gemcitabine alone [15]. This has since been adopted as the standard regimen in the treatment of unresectable GBC. Although several randomized clinical trials have investigated the value of adjuvant chemotherapy for biliary tract cancers, none have found a survival benefit in the intention-to-treat analysis and no adequately powered subgroup analyses for GBC have been conducted [16,17].

Guidelines for the treatment of localized GBC are mainly based on retrospective evidence and expert opinion due to the minimal availability of randomized evidence. Previous studies investigating GBC have typically been conducted in high-volume, non-Western centers and included patients with various biliary tract cancers [15,18,19]. Due to presumed different etiologies, results in GBC may differ from those in other biliary tract tumors [20].

Our objective was to investigate trends in treatment, establish prognostic factors associated with survival and identify opportunities for improvement in treatment stratified for disease stage.

## 2. Methods

This is a cohort study using data from the nationwide population-based Netherlands Cancer Registry (NCR), containing information on all newly diagnosed malignancies. The NCR receives notifications from the automated pathological archive (PALGA), the nation-wide network and registry of histo- and cytopathology in the Netherlands, and is supplemented by alerts from the National Archive of Hospital Discharge Diagnosis [21]. Completeness of the registry is estimated to be at least 95% [22]. Since all data was anonymized a waiver for ethical approval was provided. The STROBE guidelines for reporting of observational studies have been followed [23]. This study was approved by the NCR ethical review board and a waiver for ethical approval was provided by the Medical Ethics Review Committee of the region Arnhem-Nijmegen (CMO A-N, nr. 2017-3912) on 27/12/2017. The study was conducted according to the Declaration of Helsinki. Anonymized patient level data are available upon request from the Netherlands Cancer Registry. The statistical code is available upon request from the corresponding author.

### 2.1. Patient Selection and Variable Definitions

Clinicopathological data on all adult patients diagnosed between 2005 and 2016 with invasive gallbladder neoplasms were extracted. The following variables were provided: age, gender, year of diagnosis, socioeconomic status (social deprivation scores based on a mean number of 4000 inhabitants per 4-digit postal codes), histopathological or clinical diagnosis, tumor histology (based on the ICD-O3 classification, morphological codes are provided in Appendix A), clinical and pathological TNM stage (AJCC staging system, version 6 for patients diagnosed from 2005 to 2009 and version 7 from 2010 to 2016 [24,25]), presence and location of metastatic disease, occurrence of syn- or metachronous primary tumors, type of resection performed, resection margin (R0: microscopically free of tumor, R1 microscopically positive for tumor, R2: macroscopically positive for tumor), systemic therapy (yes/no), radiation therapy (yes/no), and duration of follow-up in days from date of diagnosis. Missing data occurred in four out of nine baseline variables (2% to 29%) and was not imputed because it was determined not to be missing at random.

Primary radical/extended cholecystectomy was defined as cholecystectomy with en-bloc excision of the gallbladder bed and dissection of the hepatoduodenal lymph nodes as the first surgery received by the patient. Re-resection was defined as any surgery for GBC after initial cholecystectomy alone within 180 days of diagnosis. Radicality was classified into R0 (resection margin microscopically free of tumor) and R1/2 (resection margin micro- or macroscopically positive). Supportive therapy included endoscopic procedures, biliary drainage and metastasectomy. Ninety-day mortality was defined as death within 90 days of diagnosis. Chemo- and radiotherapy were defined as administration of at least one dose. Information regarding type of systemic therapy received was not available. Follow-up data on vital status (complete until February 2018) were provided by linkage to the automated Municipal Personal Records Database.

### 2.2. Quality Control and Completeness of Data Assessment

Accuracy of diagnosis and completeness of histopathological assessment was assessed by comparing data from the resected patients provided by the NCR with data extracted from the medical records available from four academic centers in the Netherlands: Radboudumc, Amsterdam University Medical Center (location AMC), Erasmus MC and Leiden University Medical Center.

### 2.3. Statistical Analysis

Characteristics were described using counts and percentages for continuous variables and means and ranges for continuous variables. χ-square testing or Fisher’s exact test, where appropriate, were used to assess differences in patient characteristics. Incidence rates were calculated per 100,000 person years and age-standardized using the European standard population. Trends in incidence were assessed by calculating the estimated annual percentage change (EAPC).

Patients were grouped according to T-stage (T1/T2 vs. T3/T4), N-stage (N0 vs. N1/N2) and resection margin (R0 vs. R1/R2 vs. Rx). For survival analyses, patients were categorized as resected, non-metastatic non-resected (i.e., inoperable patients due to comorbidities and/or locally advanced disease) or metastatic at diagnosis. To assess trends in treatment over time, patients were grouped according to period of diagnosis (Period 1; 2005–2009 and Period 2; 2010–2016; these periods coincide with the introduction of gemcitabine-cisplatin chemotherapy as standard of care for unresected BTC). A subgroup analysis in patients with early (T1b/T2) disease was conducted to assess trends in surgical treatment. Kaplan-Meier curves were used to calculate median OS. OS was defined as time in days from date of diagnosis until date of death from any cause or the date of last follow-up (February 2018). Patients alive at the last date of follow-up were censored. Cox regression analysis was used to calculate hazard ratios for potential prognostic factors. Covariates were selected based on literature and entered in the multivariable model when statistically relevant (*p* < 0.1) on univariable analysis. *p*-values < 0.05 were considered statistically significant. All tests of significance were two-tailed. Statistical analyses were conducted using the SPSS 24.0 statistical package (SPSS, Inc., Chicago, IL).

## 3. Results

### 3.1. Incidence and Patient and Tumor Characteristics

**Figure 1:**
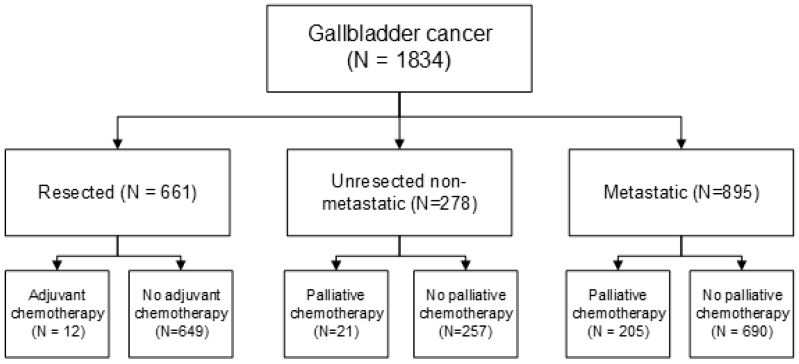
Patient flow.

Patient and tumor characteristics are shown in Table 1. Between 2005 and 2016, 1834 patients were diagnosed with GBC in the Netherlands (Figure 1). Forty-nine percent of patients had metastatic disease at diagnosis (43% from 2005 to 2009 and 53% from 2010 to 2016, *p* < 0.001). The incidence of GBC did not change significantly (EAPC—0.7%, *p* = 0.32) over time (Appendix B). Median age at diagnosis was 71 (IQR 64–80) years. Eighty percent of patients had histopathological confirmation of diagnosis.

### 3.2. Treatment

**Figure 2:**
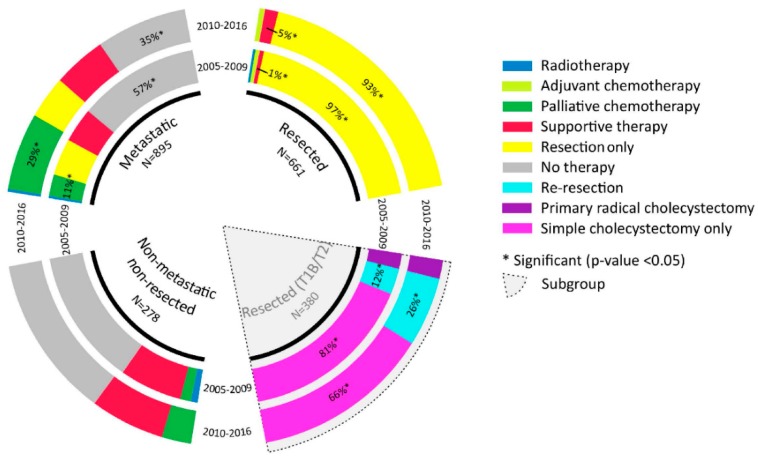
Trends in treatment in resected, non-resected non-metastatic, and metastatic gallbladder cancer (GBC). The grey area represents a subgroup analysis of resected patients with early (T1b/T2) gallbladder cancer. Percentages are only displayed when significant differences (*p* < 0.05) between periods were found. Supportive treatment includes endoscopic procedures, biliary drainage and metastasectomy.

Time trends in treatment in resected, non-resected non-metastatic, and metastatic GBC are shown in Figure 2. Among all patients with non-metastatic disease, primary resection rates increased; 64.7% in 2005 to 2009 to 74.8% in 2010 to 2016 (*p* = 0.001). More extensive tumors (T3–T4) were resected between 2010 and 2016 compared to 2009 to 2015 (from 25.1% to 33.1%, *p* < 0.001). In resected, non-metastatic patients, 90-day mortality decreased from 12.0% to 5.6% (*p* = 0.003) and the percentage of patients receiving R0 resection did not change significantly (from 70.3% to 74.7%, *p* = 0.294). The number of patients receiving an extended cholecystectomy (with/without hepatoduodenal lymphadenectomy) opposed to simple cholecystectomy in early (T1b–T2) GBC increased significantly, from 19% to 33% (*p* < 0.001). In the subgroup analysis conducted in patients with early GBC, 90-day mortality and the R0 resection rate did not change over time. Adjuvant chemotherapy was only administered to 12/661 (1.8%) patients.

Use of palliative chemotherapy did not increase in patients with unresected, non-metastatic GBC (15% vs. 15%, Figure 2). The use of palliative chemotherapy in metastatic GBC increased from 11% to 29% (*p* < 0.001).

### 3.3. Survival

**Figure 3:**
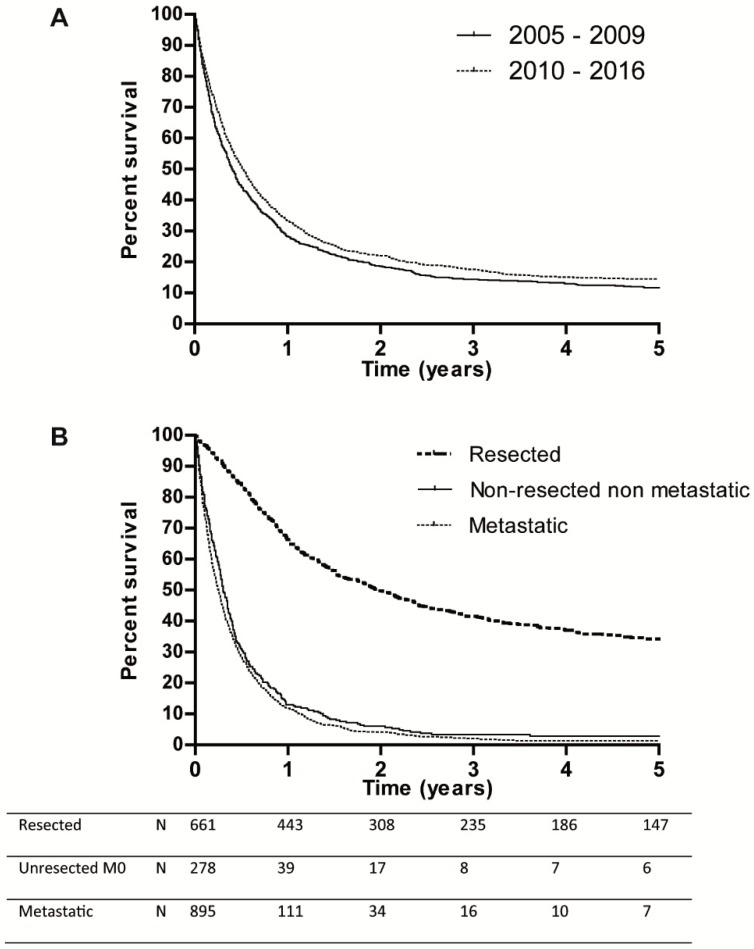
(**A**): Survival according to time period. (**B**): Survival according to disease stage.

Median OS of the entire cohort was 5.5 months (95% CI 5.0–6.0) and increased from 4.8 months (95% CI 4.2–5.4) in 2005 to 2009 to 6.1 months (95% CI 5.4–6.8) in 2010 to 2016 (*p* = 0.012) (Figure 3A). Median OS differed significantly between resected and non-metastatic non-resected/metastatic disease: 23.7 (95% CI 19.6–27.8), 3.6 (95% CI 3.1–4.6) and 2.7 (95% CI 2.6–3.2) months, respectively (*p* < 0.001, Figure 3B). Resected patients showed improved median OS over time; from 19.4 to 26.8 months (*p* = 0.038, Appendix C). Median OS in metastatic patients increased from 2.3 to 3.4 months (*p* < 0.001, Appendix C). In non-resected patients survival did not change significantly over time.

### 3.4. Therapy and Survival

Survival in patient groups with resected, non-metastatic non-resected and metastatic GBC is shown in Table 2. The survival benefit of adjuvant chemotherapy could not be assessed since only 12 out of 661 patients received some form of adjuvant therapy. Radical surgery (either primary radical cholecystectomy or re-resection) in early GBC was associated with a significantly higher median OS compared to simple cholecystectomy, from 18.4 to 76.7 months (*p* < 0.001). Palliative chemotherapy in non-resected non-metastatic and metastatic disease was associated with superior survival; from 3.5 to 7.7 (*p* = 0.011) and 2.1 versus 7.3 (*p* < 0.001) months, respectively.

### 3.5. Prognostic Factors for Survival

Poor prognostic factors were increasing age, poor tumor differentiation, higher T-stage, presence of lymph node metastases and (in resected patients) non-radical resection Table 3.

Palliative surgery and chemotherapy were associated with a better prognosis in metastatic disease (HR 0.43 and 0.47 respectively, *p* < 0.001).

### 3.6. Quality Control

In total, 108 patients (16% of resected patients) underwent a resection in one of the four academic hospitals. One patient (0.9%) turned out to have cholecystitis and was incorrectly registered by the NCR as having GBC.

## 4. Discussion

Between 2000 and 2016, no (clinically) significant changes in incidence and survival of GBC were seen. Although radical surgery in early GBC and palliative chemotherapy in unresectable and metastatic GBC significantly improved survival, these treatment modalities were only used in 33% (radical surgery) and 25% (palliative chemotherapy) of patients.

The survival rates as demonstrated in this study are comparable to those from a previously published Western cohorts [26,27], but inferior to survival rates from non-Western centers: three-year survival was 73% for stage I (53% in stage II) in our study compared to 100% (80% in stage II) in a recently conducted Korean study including 142 patients [28]. These differences are possibly attributable to selection bias in high-volume expert centers in non-Western countries, different tumor biology or differences in the administration of adjuvant chemotherapy, which has not been standard practice in the Netherlands [29].

In a subgroup analysis, improved survival over time was only seen in resected and metastatic GBC. The improved outcome of resected patients is likely the result of multiple factors. Although primary resection rates remained stable, larger tumors (T3/T4) were increasingly resected and 90-day mortality decreased significantly over time, suggesting an improvement in operative techniques or postoperative care. A sharp increase in re-resection rates for early GBC was seen after 2010, coinciding with a change in national guidelines advocating for the use of additional gallbladder bed resection and regional lymphadenectomy in early (pT1b/T2) GBC, which is associated with significantly improved outcomes [14,30,31,32,33]. Our results support this notion; patients with early GBC who received radical surgery had a median OS that was over three times larger (76.7 vs. 18.4 months) than the survival of patients who did not undergo radical resection.

Unfortunately, our results suggest substantial undertreatment; even during the last study period only 33% of patients with early-stage GBC received the recommended radical surgery in addition to cholecystectomy alone. Most likely, the majority of the early GBC patients are diagnosed incidentally after cholecystectomy for suspected benign gallbladder disease by a general gastrointestinal surgeon in a community hospital. We hypothesize that many clinicians still perceive advanced GBC as an untreatable disease and thus may be reluctant to refer patients to a specialized hepatobiliary center for additional surgery or chemotherapy. We believe that multidisciplinary, specialized care, and better adherence to (inter-)national guidelines may improve prognosis of GBC patients.

Previous studies show conflicting results on the value of adjuvant chemotherapy. Most evidence is based on small, retrospective series and only one recently published phase-3 trial showed a survival benefit in the per-protocol analysis alone [17]. Currently, recruiting large, prospective trials may show more positive results [34]. Unfortunately, the effect of adjuvant therapy after resection could not be assessed as adjuvant therapy is currently not standard of care in the Netherlands and was only administered to a small number of cases (most likely in a clinical trial setting).

In 2010, the ABC-02 trial demonstrated a survival benefit of gemcitabine and cisplatin in metastatic biliary tract cancer [15], resulting in an update of the national guidelines and palliative chemotherapy becoming standard of care. Although a subsequent rise from 15% to 25% in the use of palliative chemotherapy was seen after 2010, it was still infrequently administered. Since (subsidized) healthcare insurance is mandatory for all inhabitants of the Netherlands and travel distance to healthcare is generally short, the most likely explanation for this poor delivery rate is nihilism regarding the efficacy of chemotherapy. Evidently, chemotherapy in non-resectable GBC warrants further attention since the increase in use of palliative chemotherapy is a likely cause for the (minor) improvement in median OS in metastatic GBC.

The major limitation of this study pertains to the nature of registration data; because of the retrospective nature of this study, selection bias is present. Caution should be exercised when interpreting results, especially when analyzing treatment strategies and associated differences in survival. Additionally, possible incompleteness of data in the earlier years and changes in registry guidelines resulted in missing data on prognostic factors such as T- and N-stage (16%) in unresected patients and tumor grade (29%) in resected patients.

Second, distinguishing GBC from perihilar cholangiocarcinoma (proximal extrahepatic cholangiocarcinoma, pCC) is challenging in locally advanced disease [35]. Diagnosis in unresected patients was based on imaging only and histopathological confirmation was available in 76% of patients. However, recent research highlights the importance of this distinction, as GBC and pCC show different molecular landscapes and consequently might benefit from different treatment options [20,36,37]. The results from this study reflect current clinical practice until more reliable diagnostic methods to differentiate between GBC and pCC become available.

A unique strength of this study is the nation-wide, population based design resulting in an accurate representation of treatment and survival patterns of gallbladder cancer in daily clinical practice in a low incidence population. In addition, we were able to perform a quality control and demonstrated that the accuracy of the registration data is very high, since only 1 out of 108 patients received an incorrect diagnosis.

## 5. Conclusions

In conclusion, survival of GBC is poor and minimal improvement has been made in the past decade in the Netherlands. Radical surgery in early GBC and palliative chemotherapy in unresectable and metastatic GBC are associated with increased OS. However, the use of these treatment modalities is still limited. A multidisciplinary approach in GBC involving radical surgery and systemic therapy may lead to improvement in the survival of GBC patients.

## Figures and Tables

**Table 1:** Characteristics of patients with gallbladder cancer in the Netherlands (2005–2016).

Cohort	Total (*n* = 1834)	Resected (*n* = 661)	Non-resected Non-metastatic (*n* = 278)	Metastatic (*n* = 895)
Patient and tumor characteristics				
Age	71.1 (22–97)	69.2 (27–97)	74.3 (32–95)	71.2 (22–96)
Male sex	545 (29.1%)	206 (31.2%)	82 (29.5%)	250 (27.9%)
Socioeconomic Status				
High	501 (26.8%)	183 (27.7%)	82 (29.5%)	229 (33.4%)
Medium	741 (39.6%)	253 (38.3%)	110 (39.6%)	367 (41.0%)
Low	630 (33.7%)	225 (34.0%)	86 (30.9%)	299 (33.4%)
Clinicopathologic T stage ^a^				
T1	526 (28.1%)	147 (22.6%)	1 (0.4%)	54 (8.5%)
T2		303 (45.8%)	0 (0.0%)	22 (2.5%)
T3/T4	643 (34.3%)	172 (26.2%)	169 (60.8%)	427 (47.7%)
TX	496 (26.5%)	38 (5.8%)	13 (4.7%)	302 (33.7%)
Unknown/missing	207 (11.1%)	-	95 (34.2%)	90 (10.1%)
Clinicopathologic N stage ^a^				
N0	674 (36.0%)	140 (21.2%)	62 (22.3%)	237 (26.5%)
N1	432 (23.1%)	123 (18.6%)	74 (26.6%)	331 (37.0%)
NX	559 (29.9%)	387 (58.5%)	47 (16.9%)	237 (26.5%)
Unknown/missing	207 (11.1%)	11 (1.7%)	95 (34.2%)	90 (10.1%)
Location synchronous metastases				
Liver	N/A	N/A	N/A	350 (39.1%)
Peritoneal	N/A	N/A	N/A	119 (13.3%)
Lymph node	N/A	N/A	N/A	46 (5.1%)
Lung	N/A	N/A	N/A	11 (1.2%)
Liver + peritoneum	N/A	N/A	N/A	92 (10.3%)
Other	N/A	N/A	N/A	22 (2.5%)
Multiple, other	N/A	N/A	N/A	175 (19.6%)
Unknown/missing	N/A	N/A	N/A	80 (8.9%)
Pathology confirmation of primary tumor(yes)	1566 (83.7%)	661 (100%)	156 (56.1%)	732 (81.8%)
Differentiation grade				
Well	N/A	102 (15.4%)	N/A	N/A
Moderate	N/A	209 (31.6%)	N/A	N/A
Poor	N/A	157 (23.7%)	N/A	N/A
Not determined	N/A	193 (29.2%)	N/A	N/A
Radicality				
R0	N/A	417 (63.1%)	N/A	N/A
R1	N/A	130 (19.7%)	N/A	N/A
R2	N/A	24 (3.6%)	N/A	N/A
Unclear	N/A	90 (13.6%)	N/A	N/A

^a^ Clinical P- and N- for unresected patients and pathologic T- and N- stage for resected patients are provided.

**Table 2:** Survival of patients with gallbladder cancer according to clinical stage and treatment strategy.

Group	N	Five-year Survival	Median OS, Months (95% CI)	Log Rank Test *p* Value
Total	1895	13.2%	5.5 (5.0–6.0)	
Resected non-metastatic	661	34.2%	23.7 (19.6–27.8)	
Adjuvant chemotherapy	12	37.5%	29.4 (21.4–37.5)	0.521
No adjuvant chemotherapy	649	34.1%	23.7 (19.4–27.6)	
T1b/T2 tumor, no radical surgery	106	30.6%	18.3 (13.8–22.7)	<0.001
T1b/T2 tumor, radical surgery	274	52.7%	76.7 (43.0–110.3)	
Non-resected non-metastatic	278	2.9%	3.6 (3.1–4.1)	
No palliative chemotherapy	257	3.0%	3.5 (2.9–4.0)	0.011
Palliative chemotherapy	21	-	7.7 (4.5–10.8	
Metastatic	895	1.3%	2.9 (2.6–3.2)	
No palliative chemotherapy	690	0.6%	2.1 (1.9–2.4)	<0.001
Palliative chemotherapy	205	3.7%	7.3 (6.4–8.2)	

**Table 3:** Prognostic factors for patients with resected (**A**) and metastatic (**B**) gallbladder cancer.

**A. Prognostic factors for patients with resected gallbladder cancer. N = 661.**						
**Characteristic**	**Univariable Cox Regression**			**Multivariable Cox Regression**		
	**HR**	**95% CI**	***p* Value**	**HR**	**95% CI**	***p* Value**
Grade						
Well	1			1		
Moderate	1.41	1.02–1.95	0.036	1.17	0.84–1.61	0.354
Poor	2.67	1.93–3.70	<0.001	2.07	1.49–2.86	<0.001
Unknown	1.45	1.05–1.99	0.023	1.74	1.26–2.41	0.001
Sex						
Female	1					
Male	0.88	0.71–1.08	0.214			
Pathological T stage						
T1	1			1		
T2	1.77	1.35–2.32	<0.001	1.58	1.19–2.10	0.001
T3/T4	3.59	2.69–4.78	<0.001	2.61	1.89–3.61	<0.001
Tx	3.23	2.01–5.18	<0.001	2.16	1.34–3.50	0.002
Pathological N stage						
N0	1			1		
N1	2.96	2.13–4.12	<0.001	1.95	1.39–2.74	<0.001
Nx	2.48	1.86–3.31	<0.001	1.86	1.46–2.66	<0.001
Radicality						
R0	1			1		
R1/R2	3.78	3.03–4.71	<0.001	2.69	2.11–3.43	<0.001
Unclear	1.60	1.20–2.14	0.001	1.48	1.10–1.98	0.009
Adjuvant chemotherapy (yes)	0.67	0.33–1.36	0.268			
Prior malignancy (yes)	1.22	0.93–1.61	0.150			
Increasing age (years)	1.04	1.03–1.05	<0.001	1.04	1.03–1.05	<0.001
**B. Prognostic factors for patients with metastatic gallbladder cancer. N = 895.**						
**Characteristic**	**Univariable Cox Regression**			**Multivariable Cox Regression**		
	**HR**	**95% CI**	***p* Value**	**HR**	**95% CI**	***p* Value**
Grade						
Well	1					
Moderately	1.02	0.61–1.71	0.931			
Poor	1.45	0.89–2.36	0.136			
Unknown	1.85	1.16–2.97	0.010			
Sex						
Female	1					
Male	0.88	0.71–1.08	0.214			
Clinical T stage						
T1/T2	1			1		
T3/T4	2.01	1.57–2.58	<0.001	1.33	1.02–1.73	0.036
Tx	1.82	1.41–2.35	<0.001	1.33	1.02–1.74	0.035
Unknown	3.94	2.88–5.39	<0.001	2.22	1.57–3.15	<0.001
Clinical N stage						
N0	1			1		
N1	1.28	1.07–1.50	0.006	1.21	1.02–1.44	0.031
Nx	1.50	1.25–1.80	<0.001	1.54	1.28–1.86	<0.001
Unknown	2.70	2.11–3.47	<0.001	**		
Supportive therapy (yes)	1.07	0.90–1.27	0.443			
Palliative chemotherapy (yes)	0.46	0.39–0.54	<0.001	0.47	0.39–0.55	<0.001
Prior malignancy (yes)	0.93	0.80–1.08	0.358			
Increasing age (year)	1.03	1.03–1.04	<0.001	1.02	1.01–1.03	<0.001

** Removed due to collinearity.

## Appendix A

**ICD-03 code**		**Frequency *(%)***
8000	Neoplasma	310 *(16.6)*
8001	Tumor cells	2 *(0.1)*
8010	Carcinoma, NOS	50 *(2.7)*
8012	Large cell carcinoma NOS	35 *(1.9)*
8013	Large cell neuroendocrine carcinoma	6 *(0.3)*
8020	Carcinoma, undifferentiated, NOS	5 *(0.3)*
8030	Giant cell and spindle cell carcinoma	2 *(0.1)*
8032	Spindle cell carcinoma, NOS	2 *(0.1)*
8033	Pseudosarcomatous carcinoma	3 *(0.2)*
8041	Small cell carcinoma, NOS	10 *(0.5)*
8046	Non-small cell carcinoma	4 *(0.2)*
8070	Squamous cell carcinoma, NOS	19 *(1.0)*
8071	Squamous cell carcinoma, keratinizing, NOS	2 *(0.1)*
8074	Squamous cell carcinoma, spindle cell	1 *(0.1)*
8140	Adenocarcinoma, NOS	1171 *(62.6)*
8144	Adenocarcinoma, intestinal type	21 *(1.1)*
8160	Cholangiocarcinoma	6 *(0.3)*
8163	Pancreatobiliary-type carcinoma	5 *(0.3)*
8210	Adenocarcinoma in adenomatous polyp	7 *(0.4)*
8211	Tubular adenocarcinoma	2 *(0.1)*
8240	Carcinoid tumor, NOS	13 *(0.7)*
8244	Mixed adenoneuroendocrine carcinoma	2 *(0.1)*
8246	Neuroendocrine carcinoma, NOS	6 *(0.3)*
8249	Atypical carcinoid tumor	2 *(0.1)*
8260	Papillary adenocarcinoma, NOS	36 *(1.9)*
8263	Adenocarcinoma in tubolovillous adenoma	3 *(0.2)*
8310	Clear cell adenocarcinoma, NOS	3 *(0.2)*
8312	Renal cell carcinoma, NOS	1 *(0.1)*
8350	Nonencapsulated sclerosing carcinoma	1 *(0.1)*
8480	Mucinous adenocarcinoma	31 *(1.7)*
8481	Mucin-producing adenocarcinoma	44 *(2.4)*
8490	Signet ring cell carcinoma	19 *(1.0)*
8500	Infiltrating duct carcinoma, NOS	2 *(0.1)*
8503	Intraductal papillary adenocarcinoma with invasion	4 *(0.2)*
8560	Adenosquamous carcinoma	26 *(1.4)*
8570	Adenocarcinoma with squamous metaplasia	1 *(0.1)*
8574	Adenocarcinoma with neuroendocrine differentiation	10 *(0.5)*
8575	Metaplastic carcinoma, NOS	1 *(0.1)*
8576	Hepatoid adenocarcinoma	1 *(0.1)*
8980	Carcinosarcoma, NOS	3 *(0.2)*
